# Integrated safety and efficacy analysis of once-daily fluticasone furoate for the treatment of asthma

**DOI:** 10.1186/s12931-016-0473-x

**Published:** 2016-11-24

**Authors:** Paul M. O’Byrne, Loretta Jacques, Caroline Goldfrad, Namhee Kwon, Michael Perrio, Louisa J. Yates, William W. Busse

**Affiliations:** 1 Michael G. DeGroote School of Medicine, McMaster University, Hamilton, ON Canada; 2 Respiratory Medicine Development Centre, GSK, London, TW8 9GS UK; 3 Quantitative Sciences Division, GSK, London, TW8 9GS UK; 4 Respiratory Franchise Medical, GSK, London, TW8 9GS UK; 5 Global Clinical Safety and Pharmacovigilance, GSK, TW8 9GS London, UK; 6 University of Wisconsin School of Medicine and Public Health, Madison, WI USA; 7 Department of Medicine, Michael G. DeGroote School of Medicine, McMaster University, 1280 Main Street West, Room 3W10, Hamilton, ON L8S 4K1 Canada

**Keywords:** Adverse events, Cortisol suppression, Fluticasone furoate, Forced expiratory volume in one second, Inhaled corticosteroid, Integrated analysis, Safety

## Abstract

**Background:**

Fluticasone furoate is a once-daily inhaled corticosteroid. This report provides an overview of safety and efficacy data that support the use of once-daily fluticasone furoate 100 μg or 200 μg in adult and adolescent asthma patients.

**Methods:**

Fourteen clinical studies (six Phase II and eight Phase III) were conducted as part of the fluticasone furoate global clinical development programme in asthma. Safety data from 10 parallel-group, randomised, double-blind Phase II and III studies (including 3345 patients who received at least one dose of fluticasone furoate) were integrated to provide information on adverse events, withdrawals, laboratory assessments, vital signs and hypothalamic-pituitary-adrenal axis function. The efficacy of once-daily fluticasone furoate was evaluated in all included studies.

**Results:**

Once-daily fluticasone furoate 100 μg and 200 μg safety profiles were consistent with those reported for other inhaled corticosteroids, and both doses consistently demonstrated efficacy versus placebo. In the integrated analysis, no dose-response relationship was observed for the overall incidence of adverse events and there were no significant effects of fluticasone furoate on hypothalamic-pituitary-adrenal axis function.

**Conclusion:**

Once-daily fluticasone furoate 100 μg and 200 μg had acceptable safety profiles and was efficacious in adult and adolescent patients with asthma. There was no evidence of cortisol suppression at studied doses.

**Trial registrations:**

GSK (NCT01499446/FFA20001, NCT00398645/FFA106783, NCT00766090/112202, NCT00603746/FFA109684, NCT00603278/FFA109685, NCT00603382/FFA109687, NCT01436071/115283, NCT01436110/115285, NCT01159912/112059, NCT01431950/114496, NCT01165138/HZA106827, NCT01086384/106837, NCT01134042/HZA106829 and NCT01244984/1139879).

**Electronic supplementary material:**

The online version of this article (doi:10.1186/s12931-016-0473-x) contains supplementary material, which is available to authorized users.

## Background

Inhaled corticosteroids (ICS) are the mainstay of treatment for all severities of persistent asthma, leading to improved symptom control, improved lung function and quality of life, and reduced asthma mortality [1]. Nevertheless, poorly controlled asthma remains prevalent [2], and poor treatment adherence is well documented [1]. Most ICS are administered twice daily; however, improved compliance with once-daily regimens has been demonstrated in mild-to-moderate asthma [3]. A case-control study using the UK General Practice Research Database reported better adherence and reduced costs for patients with asthma who switched to once-daily ICS from twice-daily ICS [4].

The tolerability profile of ICS for asthma treatment is well established. Localised effects, e.g., dysphonia and oral candidiasis, have been reported [1], and prolonged high-dose ICS usage has been associated with adrenal suppression, cataracts and glaucoma [1]. Therefore, for a new ICS it is important to assess the safety profile, including potential systemic effects, e.g., cortisol supression.

The ICS fluticasone furoate (FF), administered using the ELLIPTA® dry powder inhaler, is a once-daily maintenance treatment for adult and adolescent (≥12 years) patients with asthma. FF has higher cellular accumulation and slower efflux compared with other corticosteroids [5], resulting in enhanced lung residency and 24-h efficacy in patients with asthma [6–8]. FF 100 μg or 200 μg is approved as monotherapy in a number of countries, including the USA [9], and in combination with vilanterol, a novel long-acting beta_2_ agonist, as asthma maintenance therapy in multiple countries, including the USA and in the EU [10–12].

An integrated safety analysis and overview of efficacy data from Phase II and III studies, which assessed the overall benefit and risks of once-daily FF 100 μg or 200 μg for patients with varying severities of persistent asthma, was carried out using all available data.

## Methods

### Clinical studies

Fourteen clinical studies (six Phase II and eight Phase III) were conducted (FF asthma global clinical development programme): 10 assessed FF only and four investigated FF/vilanterol with a FF arm (Table 1 [13–26]). In 12 studies, FF was administered using the ELLIPTA® dry powder inhaler in the evening. The remaining studies used Rotadisk Diskhaler™ [13] or Diskus™/Accuhaler™ [14], in the morning or evening.

**Table 1:** Clinical studies conducted as part of the FF global clinical development programme in asthma

Study	Phase	Number of patients	Treatment arms	Study duration, weeks	Primary end-point	Additional end-points	Included in integrated safety analysis
Medley et al. [13]^a^ (NCT01499446)	II dose regimen	575	FF 100 μg OD morning; FF 100 μg OD evening; FF 250 μg evening; placebo	4	Mean change from baseline in daily trough PEF	FEV_1_, symptom-free days, rescue-free days, withdrawals, safety (AEs, 24-h UFC)	No
Woodcock et al. [14]^b^ (NCT00398645)	II dose regimen	545	FF 200, 400 μg OD morning; FF 200, 400 μg OD evening; FF 200 μg BD; placebo	8	Mean change from baseline in trough FEV_1_	Safety (AEs, withdrawals, 24-h UFC)	No
Woodcock et al. [15]^c^ (NCT00766090)	II dose regimen	190	FF, FP 200 μg OD evening; FF, FP 100 μg BD; placebo	4	Mean change from baseline in trough FEV_1_	Safety (AEs, 24-h UFC)	No
Busse et al. [16]^c^ (NCT00603746)	II dose ranging	627	FF 200, 400, 600, 800 μg OD evening; FP 500 μg BD; placebo	8	Mean change from baseline in trough FEV_1_	Asthma symptom scores, PEF, symptom-free days, rescue-free days, withdrawals, safety (AEs, 24-h UFC)	Yes
Bleecker et al. [17]^c^ (NCT00603278)	II dose ranging	622	FF 100, 200, 300, 400 μg OD evening; FP 250 μg BD; placebo	8	Mean change from baseline in trough FEV_1_	PEF, symptom-free and rescue-free periods, withdrawals, safety (AEs, 24-h UFC)	Yes
Bateman et al. [18]^c^ (NCT00603382)	II dose ranging	598	FF 25, 50, 100, 200 μg OD evening; FP 100 μg BD; placebo	8	Mean change from baseline in trough FEV_1_	PEF, symptom-free and rescue-free periods, withdrawals, safety (AEs, 24-h UFC)	Yes
O’Byrne et al. [19]^c^ (NCT01436071)	III efficacy	248	FF 50 μg OD evening; placebo	12	Mean change from baseline in trough FEV_1_	Rescue-free and symptom-free 24-h periods, PEF, ACT, QoL, safety (AEs, severe exacerbations)	Yes
Busse et al. [20]^c^ (NCT01436110)	III efficacy	351	FF 50 μg OD evening; FP 100 μg BD; placebo	24	Mean change from baseline in trough evening FEV_1_	Rescue-free and symptom-free 24-h periods, PEF, ACT, QoL, safety (AEs, severe exacerbations)	Yes
Lötvall et al. [21]^c^ (NCT01159912)	III efficacy	343	FF 100 μg OD evening; FP 250 μg BD; placebo	24	Mean change from baseline in trough evening FEV_1_	Rescue-free and symptom-free 24-h periods, PEF, ACT, QoL, safety (AEs, severe exacerbations, 24-h UFC)	Yes
Woodcock et al. [22]^c^ (NCT01431950)	III efficacy	238	FF 100 μg, 200 μg OD evening	24	Mean change from baseline in trough FEV_1_	Rescue-free and symptom-free 24-h periods, PEF, ACT, safety (AEs, severe exacerbations, 24-h UFC)	Yes
Bleecker et al. [23]^c^ (NCT01165138)	III efficacy	609	FF/VI 100/25 μg; FF 100 μg OD evening; placebo	12	Mean change from baseline in trough FEV_1_ and serial (0–24 h) weighted mean FEV_1_	Rescue-free and symptom-free 24-h periods, QoL, withdrawals, safety (AEs, severe exacerbations, 24-h UFC)	Yes
Bateman et al. [24]^c^ (NCT01086384)	III efficacy	2019	FF/VI 100/25 μg, FF 100 μg OD evening	≥24–78	Time to first severe exacerbation	Rate of severe exacerbations per patient per year, trough FEV_1_, safety (hospitalisations, AEs)	Yes
O’Byrne et al. [25]^c^ (NCT01134042)	III efficacy	586	FF/VI 200/25 μg OD evening; FF 200 μg OD evening; FP 500 μg BD	24	Mean change from baseline in trough FEV_1_ and serial (0–24 h) weighted mean FEV_1_	Rescue-free 24-h and symptom-free 24-h periods, QoL, PEF, ACT, safety (AEs, 24-h UFC)	Yes
Muraki et al. [26]^c^ (NCT01244984)	III safety	243	FF/VI 100/25 μg, FF/VI 200/25 μg, FF 100 μg OD evening	52	Safety (AEs, severe exacerbations, 24-h UFC)	PEF, asthma symptom scores	No

Clinicaltrials.gov study registration numbers are provided in brackets after each study citation

FF/VI 100/25 μg = 92/22 μg (emitted). FF/VI 200/25 μg = 184/22 μg (emitted). FF 100 μg = 90 μg (emitted). FF 200 μg = 182 μg (emitted)

*ACT* asthma control test^TM^, *AE* adverse event*, BD* twice daily, *FEV*_*1*_ forced expiratory volume in one second, *FF* fluticasone furoate, *FP* fluticasone propionate, *OD* once daily, *PEF* peak expiratory flow, *QoL* quality of life, *UFC* urinary free cortisol excretion, *VI* vilanterol

^a^FF administered via Rotadisk Diskhaler^TM^

^b^FF administered via Diskus^TM^/Accuhaler^TM^

^c^FF administered via ELLIPTA® inhaler

Across the 14 studies, inclusion criteria were as follows: patients ≥12 years of age; a clinical history of asthma (in accordance with the definition of National Institutes of Health [27]); forced expiratory volume in one second (FEV_1_), 40–90% of the predicted normal value (50–90% in one study [24]); bronchodilator reversibility of disease (≥12% and ≥200 mL increase in FEV_1_ within 10–40 min following two to four inhalations of albuterol/salbutamol); and documented use of albuterol/salbutamol and/or asthma symptoms on ≥4 of the last 7 consecutive days of a run-in period (or in one study, ≥3 of the last 7 consecutive days of the run-in period [24]). As patients entering the Phase III studies were symptomatic on ICS or ICS/LABA therapy, their asthma severity may be considered equivalent to GINA steps 3–4 [1].

All studies complied with the principles of Good Clinical Practice [28] and were approved by relevant Ethics Committees/Institutional Review Boards. Written informed consent was obtained. Studies were conducted in accordance with the applicable version of the Declaration of Helsinki [29]. Regulatory approval was obtained from the relevant health authority where required.

### Safety analysis

Key comparisons of interest for integrated safety analyses were FF 100 μg versus placebo and FF 200 μg versus placebo. Data from 10 completed Phase II and III parallel-group, double-blind studies, in which FF was delivered via the ELLIPTA® inhaler, were integrated to assess FF safety (Table 1). The four additional studies provided further supporting safety data for FF [13–15, 26]; however, these were reviewed individually and not integrated as they had either a different design (e.g., crossover/open-label) or did not use the ELLIPTA® inhaler. The treatment groups analysed were as follows: once-daily FF 50 μg, 100 μg and 200 μg; twice-daily fluticasone propionate (FP) 100 μg, 250 μg and 500 μg; and placebo. All randomised patients who received at least one dose of study medication were included in the integrated analysis. As the data were integrated to support the regulatory filings of FF monotherapy for the treatment of asthma, the cut-off for this analysis was 15 February 2013; data from studies reported following this date were not included.

The integrated analysis determined (a) the rate of adverse events (AEs) and (b) the exposure-adjusted AE rate (to account for variation in treatment exposure across the groups) reported as the number of patients with an event per 1000 patient-years of exposure.

AEs were coded and grouped by System Organ Class and Preferred Term using the Medical Dictionary for Regulatory Activities (MedDRA, version 15.1). AEs of special interest (AESI) were defined using pre-selected MedDRA preferred terms and based on the known AE profile/pharmacology of corticosteroids: hypersensitivity, bone disorders, local steroid effects (e.g., oral candidiasis/hoarseness), ocular effects, glucose effects, pneumonia, lower respiratory tract infection and systemic effects (e.g., hypothalamic-pituitary-adrenal [HPA] axis). A severe asthma exacerbation was defined as an asthma deterioration requiring the use of systemic or oral corticosteroids for ≥3 days, or an in-patient hospitalisation or emergency department visit due to asthma that required systemic corticosteroids (asthma exacerbations were only recorded as AEs if they met the definition of a serious AE). Vital signs (diastolic and systolic blood pressure, heart rate) and laboratory assessments (clinical chemistry and haematology parameters) were also integrated.

The asthma clinical programme assessed 24-h urine and serum cortisol excretion. Urinary cortisol measurements were collected in seven of the 10 studies integrated for safety. Urinary cortisol excretion was log-transformed and analysed in the urine cortisol population (a subset of patients whose urine samples did not have confounding factors that could affect the interpretation of the results, e.g., inadequate urine volume, inappropriate collection time, 24-h creatinine excretion below the lower limit of threshold, use of prohibited medications or missing the baseline and/or end of treatment assessments). An analysis of covariance model was used, controlling for the following baseline effects (log): region, sex, age, treatment and study.

### Efficacy analysis

Three strengths of FF monotherapy were assessed as part of a Phase III programme [19–26]: FF 50 μg, 100 μg and 200 μg (Table 1). The efficacy of FF 50 μg was not demonstrated and was not submitted for regulatory approval. The efficacy assessment mainly used data from individual studies. Trough (24-h post-dose for once-daily FF) FEV_1_, serial 0–24-h weighted mean FEV_1_ and peak expiratory flow (PEF) were evaluated, as well as symptomatic end-points (rescue-free and symptom-free 24-h periods) and Asthma Control Test™ score. To support regulatory submissions, the only two Phase III studies that were placebo controlled and included FF 100 μg were integrated.

Access to the datasets supporting the conclusions of this manuscript may be obtained via https://www.clinicalstudydatarequest.com/.

## Results

A total of 4203 patients in the 10 studies that were integrated for the safety analysis received at least one dose of study drug (3345 patients received at least one dose of FF). Treatment exposure varied across treatment groups from 60.28 to 1179.36 patient-years, with the greatest exposure reported for the FF 100 μg group (Additional file 1: Table S1). As a placebo comparator is not ethical in longer-term studies, a placebo arm was not included in studies longer than 24 weeks. A total of 537 patients (32%) in the FF 100 μg group were treated for >52 weeks.

### Safety

A summary of the AE incidence and exposure-adjusted AE incidence for each treatment arm is provided (Table 2). The most frequently reported AEs for the FF treatment groups in the integrated analysis were headache, nasopharyngitis, upper respiratory tract infection, bronchitis, oropharyngeal pain and cough (Table 3). There were no additional findings in the non-integrated studies.

**Table 2:** Summary of the AE profile for the integrated clinical studies

AE (preferred term), *n* (%)	Placebo (*n* = 858)	FF 50 μg OD (*n* = 338)	FF 100 μg OD (*n* = 1663)	FF 200 μg OD (*n* = 608)	FP 100 μg BD (*n* = 217)	FP 250 μg BD (*n* = 214)	FP 500 μg BD (*n* = 305)
Any AE	278 (32)	121 (36)	912 (55)	256 (42)	94 (43)	90 (42)	136 (45)
Any drug-related AE	22 (3)	8 (2)	103 (6)	36 (6)	10 (5)	18 (8)	24 (8)
Any SAE	7 (<1)	1 (<1)	38 (2)	7 (1)	3 (1)	1 (<1)	4 (1)
Any drug-related SAE	0	0	3 (<1)	0	0	0	1 (<1)
Any AE leading to discontinuation of study drug	8 (<1)	2 (<1)	28 (2)	10 (2)	4 (2)	4 (2)	6 (2)
Deaths	0	0	2 (<1)^a^	0	0	0	0

*AE* adverse event, *BD* twice daily, *FF* fluticasone furoate, *OD* once daily, *FP* fluticasone propionate, *SAE* serious adverse event

^a^Neither death was determined by the investigator to be related to study medication

**Table 3:** Most frequent on-treatment AEs reported with ≥ 3% incidence in any treatment groups (integrated clinical studies)

AE (preferred term), *n* ^a^ (%)	Placebo (*n* = 858)	FF 50 μg OD (*n* = 338)	FF 100 μg OD (*n* = 1663)	FF 200 μg OD (*n* = 608)	FP 100 μg BD (*n* = 217)	FP 250 μg BD (*n* = 214)	FP 500 μg BD (*n* = 305)
Any AE	278 (32)	121 (36)	912 (55)	256 (42)	94 (43)	90 (42)	136 (45)
Headache	66 (8)	29 (9)	228 (14)	44 (7)	24 (11)	15 (7)	25 (8)
Nasopharyngitis	45 (5)	15 (4)	181 (11)	53 (9)	14 (6)	11 (5)	43 (14)
URTI	16 (2)	8 (2)	111 (7)	15 (2)	7 (3)	12 (6)	7 (2)
Bronchitis	15 (2)	0	98 (6)	15 (2)	3 (1)	5 (2)	7 (2)
Oropharyngeal pain	11 (1)	2 (<1)	71 (4)	19 (3)	4 (2)	6 (3)	11 (4)
Cough	9 (1)	3 (<1)	68 (4)	13 (2)	2 (<1)	5 (2)	15 (5)
Pharyngitis	24 (3)	14 (4)	55 (3)	8 (1)	5 (2)	2 (<1)	7 (2)
Sinusitis	8 (<1)	5 (1)	53 (3)	15 (2)	6 (3)	5 (2)	6 (2)
Influenza	9 (1)	5 (1)	45 (3)	17 (3)	6 (3)	0	7 (2)
Back pain	4 (<1)	7 (2)	52 (3)	11 (2)	4 (2)	2 (<1)	4 (1)
Dysphonia	4 (<1)	1 (<1)	23 (1)	11 (2)	3 (1)	6 (3)	6 (2)
Rhinitis	7 (<1)	1 (<1)	27 (2)	7 (1)	3 (1)	0	8 (3)
Viral respiratory tract infection	0	2 (<1)	18 (1)	8 (1)	0	0	8 (3)
	Exposure-adjusted incidence rate per 1000 patient-years						
AE (preferred term), *n* (%)	Placebo	FF 50 μg OD	FF 100 μg OD	FF 200 μg OD	FP 100 μg BD	FP 250 μg BD	FP 500 μg BD
Patient-years	185.6	87.5	1179.4	169.2	61.0	60.3	95.7
Headache	355.6	331.5	193.3	260.1	393.4	248.8	261.2
Nasopharyngitis	242.5	171.5	153.5	313.3	229.5	182.5	449.3
URTI	86.2	91.4	94.1	88.7	114.8	199.1	73.1
Bronchitis	80.8	0	83.1	88.7	49.2	82.9	73.1
Oropharyngeal pain	59.3	22.9	60.2	112.3	65.6	99.5	114.9
Cough	48.5	34.3	57.7	76.9	32.8	82.9	156.7
Pharyngitis	129.3	160.0	46.6	47.3	82.0	33.2	73.1
Sinusitis	43.1	57.2	44.9	88.7	98.4	82.9	62.7
Influenza	48.5	57.2	38.2	100.5	98.4	0	73.1
Back pain	21.6	80.0	44.1	65.0	65.6	33.2	41.8
Dysphonia	21.6	11.4	19.5	65.0	49.2	99.5	62.7
Rhinitis	37.7	11.4	22.9	41.4	49.2	0	83.6
Viral respiratory tract infection	0	22.9	15.3	47.3	0	0	83.6

*AE* adverse event, *BD* twice daily, *FF* fluticasone furoate, *FP* fluticasone propionate, *OD* once daily, *URTI* upper respiratory tract infection

^a^Numbers represent the number of patients with an event per 1000 patient-years of exposure

Nasopharyngitis, oropharyngeal pain, sinusitis, influenza, back pain, dysphonia and viral respiratory tract infection occurred at a higher rate in patients treated with FF 100 μg and 200 μg, versus placebo. However, there were generally no differences in the exposure-adjusted AE rates for FF 100 μg versus placebo, except for back pain and viral respiratory tract infection. Similarly, there were no clear differences in exposure-adjusted AE rates between FF and FP.

In two long-term studies (FF 100 μg for >6 months [24, 26]), there was generally no difference in the pattern of new AE occurrence after 6 months and no increased incidence of AEs during the second 6 months, compared with the first 6 months. For example, in one study of up to 78 weeks, the AE incidence with an onset in the first 6 months was 54%, compared with 38% with an onset of after 6 months. There was no pattern to suggest a difference in the AE profile according to length of study medication exposure.

The most frequently reported drug-related AEs in the FF treatment groups were headache, dysphonia and oral/oropharyngeal candidiasis. The incidence of drug-related AEs ranged from 2% with FF 50 μg to 6% with FF 100 μg and 200 μg for once-daily administration, and up to 8% with twice-daily FP 250 μg and FP 500 μg.

Serious AEs (SAEs) were experienced by <1–2% of patients across the treatment groups (Table 2), most frequently in the FF 100 μg group (2%, *n* = 38). Asthma exacerbation was the most common SAE, experienced by nine patients in the FF 100 μg group and one patient in each of the placebo, FF 200 μg and twice-daily FP 500 μg groups. All nine patients with severe asthma exacerbations in the FF 100 μg groups were from a study that specifically examined exacerbations. That study was ≤78 weeks in duration (compared with ≤24 weeks in other studies), had no placebo arm and, unlike other studies, recruited patients with a history of severe asthma exacerbations in the previous year [24]. Only four SAEs were considered by the investigator to be treatment related: pneumonia, asthma exacerbation and non-cardiac chest pain with FF 100 μg, and haemoptysis with twice-daily FP 500 μg. There was no notable difference in the incidence of AEs leading to withdrawal across treatment groups (0–2%; Table 2); the most frequent of these were asthma exacerbation, dyspnoea and pneumonia.

Local steroid effects (comprising oropharyngeal pain, dysphonia, oral candidiasis and oropharyngeal candidiasis) were the most frequent AESI; the exposure-adjusted incidence per 1000 patient-years was 80.8 (placebo), 103.4 (FF 100 μg) and 283.8 (FF 200 μg; Table 4). No AEs indicative of HPA axis disorders were reported. The pneumonia incidence was low (≤0.7%) across all groups (Additional file 2: Figure S1). The exposure-adjusted incidence of pneumonia (Table 4) was similar between FF 100 μg and placebo, and was numerically higher with FF 200 μg (8.5, 10.8 and 23.6, respectively, per 1000 patient-years); however, the confidence intervals (CIs) were wide and overlapped across all groups, including placebo (Additional file 2: Figure S1). Serious pneumonia occurred at comparable rates for FF 100 μg, FF 200 μg and placebo (4.2, 5.9 and 5.4, respectively, per 1000 patient-years; Additional file 3: Figure S2).

**Table 4:** AESI occurring in any treatment groups (integrated clinical studies)

AE of special interest (preferred term), *n* ^a^ (%)	Placebo (*n* = 858)	FF 50 μg OD (*n* = 338)	FF 100 μg OD (*n* = 1663)	FF 200 μg OD (*n* = 608)	FP 100 μg BD (*n* = 217)	FP 250 μg BD (*n* = 214)	FP 500 μg BD (*n* = 305)
Local steroid effects	15 (2)	7 (2)	122 (7)	48 (8)	8 (4)	18 (8)	25 (8)
Oropharyngeal pain	11 (1)	2 (<1)	71 (4)	19 (3)	4 (2)	6 (3)	11 (4)
Dysphonia	4 (<1)	1 (<1)	23 (1)	11 (2)	3 (1)	6 (3)	6 (2)
Oral candidiasis	0	4 (1)	18 (1)	8 (1)	1 (<1)	4 (2)	4 (1)
Oropharyngeal candidiasis	1 (<1)	1 (<1)	7 (<1)	9 (1)	1 (<1)	2 (<1)	6 (2)
LRTI excluding pneumonia	16 (2)	1 (<1)	114 (7)	19 (3)	3 (1)	5 (2)	7 (2)
Bronchitis	15 (2)	0	98 (6)	15 (2)	3 (1)	5 (2)	7 (2)
Hypersensitivity^b^	13 (2)	3 (<1)	41 (2)	6 (<1)	2 (<1)	2 (<1)	6 (2)
Bone disorders^b^	0	2 (<1)	21 (1)	2 (<1)	1 (<1)	0	4 (1)
Pneumonia^b^	2 (<1)	0	10 (<1)	4 (<1)	1 (<1)	0	0
Effects on glucose^b^	0	0	11 (<1)	2 (<1)	0	0	0
Ocular effects^b^	0	0	6 (<1)	0	0	0	0
		Exposure-adjusted incidence rate per 1000 patient-years					
AE of special interest (preferred term), *n* (%)	Placebo	FF 50 μg OD	FF 100 μg OD	FF 200 μg OD	FP 100 μg BD	FP 250 μg BD	FP 500 μg BD
Patient-years	185.6	87.5	1179.4	169.2	61.0	60.3	95.7
Local steroid effects	80.8	80.0	103.4	283.8	131.1	298.6	261.2
Oropharyngeal pain	59.3	22.9	60.2	112.3	65.6	99.5	114.9
Dysphonia	21.6	11.4	19.5	65.0	49.2	99.5	62.7
Oral candidiasis	0	45.7	15.3	47.3	16.4	66.4	41.8
Oropharyngeal candidiasis	5.4	11.4	5.9	53.2	16.4	33.2	62.7
LRTI excluding pneumonia	86.2	11.4	96.7	112.3	49.2	82.9	73.1
Bronchitis	80.8	0	83.1	88.7	49.2	82.9	73.1
Hypersensitivity^b^	70.0	34.3	34.8	35.5	32.8	33.2	62.7
Bone disorders^b^	0	22.9	17.8	11.8	16.4	0	41.8
Pneumonia^b^	10.8	0	8.5	23.6	16.4	0	0
Effects on glucose^b^	0	0	9.3	11.8	0	0	0
Ocular effects^b^	0	0	5.1	0	0	0	0

*AE* adverse event, *BD* twice daily, *FF* fluticasone furoate, *FP* fluticasone propionate, *LRTI* lower respiratory tract infection, *OD* once daily

^a^Numbers represent the number of patients with an event per 1000 patient-years of exposure

^b^No individual event occurred in ≥1% of patients

**Fig. 1:**
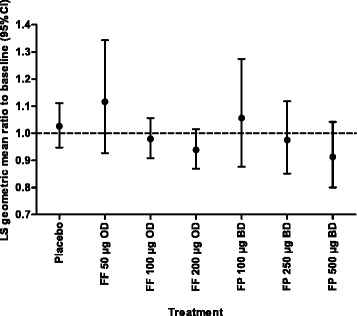
Least squares geometric mean ratio to baseline (95% CI) in urinary free cortisol excretion at end of treatment (integrated clinical studies, urine cortisol population). Analysis performed using ANCOVA with covariates of region, study, gender, age, treatment and the log of the baseline values. Includes studies NCT00603746, NCT00603278, NCT00603382, NCT01159912, NCT01431950, NCT01165138 and NCT01134042 [16–18, 21–23, 25]. Abbreviations: *ANCOVA*, analysis of covariance; *BD*, twice daily; *CI*, confidence interval; *FF*, fluticasone furoate; *FP*, fluticasone propionate; *LS*, least squares; *OD*, once daily

Urinary cortisol levels were measured in seven of the 10 integrated studies (Table 1). At the end of treatment, 24-h mean urinary cortisol excretion levels were similar to baseline levels and all mean ratios from treatment end to baseline were close to 1 in the once-daily FF 100 μg and 200 μg, twice-daily FP 100 μg, 250 μg and 500 μg, and placebo groups. In the adjusted means analysis, there were no statistically significant differences in 24-h urinary cortisol excretion from baseline to treatment end between each FF treatment group and placebo (Fig. 1); the percentage of patients with a change from normal urinary cortisol levels at baseline to low levels during treatment was similar between the FF (<1%, FF 100 μg, and 3%, FF 200 μg) and placebo (2%) treatment groups.

Vital sign and laboratory assessments were reviewed as part of the integrated analysis; no apparent treatment-related effects were observed.

## Efficacy in Phase II/III studies

### Lung function

**Fig. 2:**
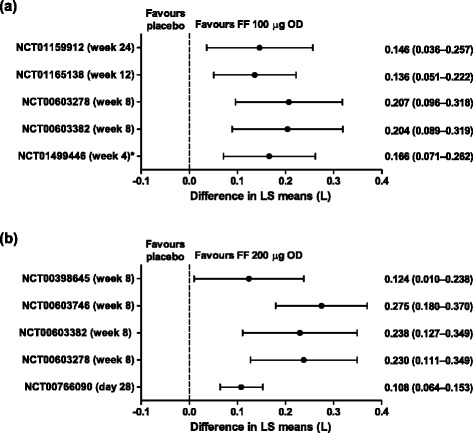
Forest plots for change from baseline in trough FEV_1_. (**a**) FF 100 μg versus placebo (ITT population), and (**b**) FF 200 μg versus placebo (ITT population). *In study NCT01499446, the final approved inhaler for FF delivery was not used [13]. Abbreviations: *FEV*_*1*_, forced expiratory volume in one second; *FF*, fluticasone furoate; *ITT*, intent-to-treat; *LS*, least squares; *OD*, once daily

Across the Phase II and III studies, FF 100 μg and 200 μg consistently improved trough FEV_1_, compared with placebo (Fig. 2). In Phase III studies, the treatment differences between FF 100 μg and placebo for change from baseline in trough FEV_1_ were 146 mL (95% CI: 36–257 [21]) and 136 mL (95% CI: 51–222 [23]), at 24 weeks and 12 weeks, respectively. In one study, weighted mean FEV_1_ (0–24 h; change from baseline) was measured and FF 100 μg demonstrated a statistically significant difference of 186 mL (95% CI: 62–310) from placebo at the end of the 12-week treatment period [23]. In another study, the efficacy of once-daily FF 200 μg was similar to twice-daily FP 500 μg in terms of trough and 0–24-h weighted mean FEV_1_ improvement [25]. Finally, in a Phase II study, patients with persistent asthma not controlled by short-acting beta_2_ agonists had significant FEV_1_ and evening PEF improvements after receiving FF 100 μg or 200 μg, versus placebo (*p* ≤0.005 [18]).

### Symptomatic end-points

**Fig. 3:**
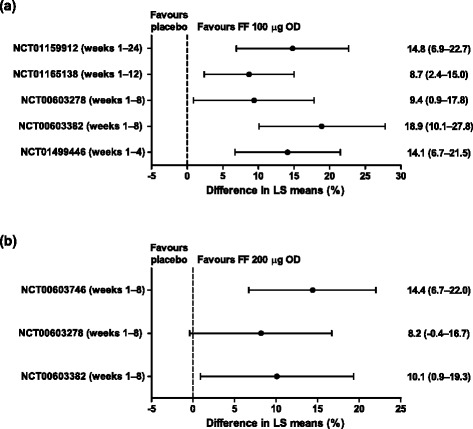
Forest plots for change from baseline (95% CI) in percentage of rescue-free 24-h periods. (**a**) FF 100 μg versus placebo (ITT population), and (**b**) FF 200 μg versus placebo (ITT population). Abbreviations: *CI*, confidence interval; *FF*, fluticasone furoate; *ITT*, intent-to-treat; *LS*, least squares; *OD*, once daily

FF 100 μg and 200 μg resulted in improvements in the proportion of rescue-free 24-h periods versus placebo in all studies where this was measured (Fig. 3). In the only two Phase III studies that compared FF with placebo, FF 100 μg demonstrated significant improvements in the proportion of rescue-free 24-h periods versus placebo (14.8%, *p* <0.001 [23], and 8.7%, *p* = 0.007 [21]).

### Exacerbations

Exacerbations were assessed in the integrated safety analysis. Patients who received FF 100 μg had a 53.4% risk reduction for a severe asthma exacerbation by the end of the treatment period, compared with placebo (hazard ratio [HR]: 0.466, 95% CI: 0.240–0.906; *p* = 0.024). Relative to placebo-treated patients, risk reductions for severe asthma exacerbations were also seen with FF 200 μg (64.8% risk reduction; HR: 0.352, 95% CI: 0.171–0.724; *p* = 0.005).

## Discussion

In this integrated analysis, FF had an acceptable safety profile, comparable with other ICS. The specific effects seen were consistent with known ICS class effects. The most frequently reported AEs were as anticipated, having been reported with other ICS (upper respiratory tract infection, bronchitis, oropharyngeal pain and cough [30]) or commonly experienced by patients with asthma (headache and nasopharyngitis were reported at similar frequencies in the placebo group). Despite a dose-response relationship between ICS use in asthma and oral candidiasis being well recognised, oral/oropharyngeal candidiasis rates were low and did not occur in more than 2% of FF- or FP-treated patients in this integrated analysis. However, the exposure-adjusted presentation of AESI indicated a higher incidence of local steroid effects for FF 200 μg, compared with FF 100 μg and placebo.

Pneumonia was pre-specified as an AESI because it has been reported as an ICS-related event in chronic obstructive pulmonary disease, but no clear association has been identified with the use of ICS and pneumonia in asthma [31]. When assessing AEs across multiple studies of varying lengths and population size, it is important to consider exposure-adjusted incidence to account for disparities. In this integrated analysis, the incidence of pneumonia was low (<1%) in all treatment groups and, although the exposure-adjusted incidence of pneumonia was slightly higher with FF 200 μg than FF 100 μg or placebo, the CIs were wide and overlapped (Additional file 2: Figure S1). An increased pneumonia incidence with higher doses cannot be ruled out, but the absolute risk of pneumonia was small and the exposure-adjusted incidence rates were consistent with the background rate in the asthmatic population [32] and, for FF 200 μg, comparable to that calculated for placebo, FP and budesonide in a meta-analysis [31]. There was no evidence of a higher rate of other AESI (hypersensitivity, bone disorders, adrenal suppression, ocular effects and effects on glucose [based on known pharmacological effects of corticosteroids]), relative to placebo or active comparator.

Beyond the present analysis, the safety profile of FF is supported by the clinical experience of FF in combination with vilanterol. Long-term safety evaluation of FF/vilanterol 100/25 μg and FF/vilanterol 200/25 μg has not identified any additional potential corticosteroid-related effects or significant findings from formal ophthalmological examinations after 52 weeks of exposure [33].

Cortisol suppression has been observed in patients with asthma who have normal HPA axis function at baseline receiving high doses of ICS [34]. However, in the studies included in our analyses there were no reports of significant cortisol suppression. In a formal HPA axis study, measurement of 24-h serum cortisol (a sensitive method for assessing adrenocortical activity) was used to assess non-inferiority of FF/vilanterol (100/25 μg and 200/25 μg) compared with placebo [35]. There were no statistically significant differences in 0–24-h weighted mean serum cortisol between either FF/vilanterol treatment and placebo after 6 weeks of treatment, but the active control (7 days of once-daily prednisolone 10 mg) significantly reduced serum cortisol levels. In the present study, few patients in any treatment group experienced below-normal levels of urinary free cortisol excretion at the end of treatment, and the incidence reported within the FF groups was similar to that in placebo.

In the efficacy analysis, once-daily FF 100 μg and 200 μg treatment for patients with persistent asthma produced consistent trough FEV_1_ improvements and increases in the proportion of rescue-free 24-h periods, compared with placebo. This present analysis examined evening dosing (primarily), but a subsequent study has suggested that morning and evening FF doses are equally efficacious as assessed by improvement from baseline in FEV_1_ versus placebo [36].

Consistent with previous results [37], the data presented here suggest that FF has a wide therapeutic index; the therapeutic dose range was efficacious across individual studies, with a tolerability profile as expected for the class and no evidence of cortisol suppression at the doses assessed.

The strengths of the analyses presented here include the large number of safety end-points assessed, the dataset size (14 studies, 6241 patients) and the length of the studies (up to 76 weeks). A limitation was the disparity in exposure across the treatment groups due to differences in population size and treatment duration, which was mitigated somewhat by the use of exposure-adjusted data.

## Conclusions

In conclusion, this integrated safety analysis demonstrates that the safety profile of FF is consistent with known ICS class effects in patients with asthma, such as local steroid effects, and once-daily FF 100 μg and FF 200 μg doses are well tolerated in adult and adolescent patients with a range of asthma severities. There was no evidence of cortisol suppression at the doses studied. It should be noted that the safety data has been pooled from different studies and that the individual studies were not designed to identify significant differences regarding specific AEs.

## Additional files

Additional file 1: Table S1. Summary of demographic characteristics and treatment exposure by treatment group (DOCX 41 kb)
Additional file 2: Figure S1. Plot of pneumonia incidence per 1000 patient-years and 95% CI by treatment group (integrated clinical studies). *BD*, twice daily; *CI*, confidence interval; *FF*, fluticasone furoate; *FP*, fluticasone propionate; *OD*, once daily. (TIF 465 kb)
Additional file 3: Figure S2. Plot of serious pneumonia incidence per 1000 patient-years and 95% CI by treatment group (integrated clinical studies). *BD*, twice daily; *CI*, confidence interval; *FF*, fluticasone furoate; *FP*, fluticasone propionate; *OD*, once daily. (TIF 427 kb)

## Abbreviations

AE: Adverse event
AESI: Adverse event of special interest
CI: Confidence interval
FEV_1_: Forced expiratory volume in one second
FF: Fluticasone furoate
FP: Fluticasone propionate
HPA: Hypothalamic-pituitary-adrenal
HR: Hazard ratio
ICS: Inhaled corticosteroids
MedDRA: Medical dictionary for regulatory activities
PEF: Peak expiratory flow
SAE: Serious adverse event
