# Supplementary material for: Integrated safety and efficacy analysis of once-daily fluticasone furoate for the treatment of asthma
Source: Respir Res. 2016 Nov 24;17:157. doi: 10.1186/s12931-016-0473-x (PMC5122018; doi:10.1186/s12931-016-0473-x)
Supplement: Additional file 1: Table S1. — Summary of demographic characteristics and treatment exposure by treatment group (DOCX 41 kb) [file 12931_2016_473_MOESM1_ESM.docx]

Additional file 1: Table S1. Summary of demographic characteristics and treatment exposure by treatment group

|  | Placebo  (*n* = 858) | FF 50 µg  OD  (*n* = 338) | FF 100 µg  OD  (*n* = 1663) | FF 200 µg  OD  (*n* = 608) | FP 100 µg  BD  (*n* = 217) | FP 250 µg  BD  (*n* = 214) | FP 500 µg  BD  (*n* = 305) |
| --- | --- | --- | --- | --- | --- | --- | --- |
| Gender, *n* (%)  Female  Male | 520 (61)  338 (39) | 201 (59)  137 (41) | 1091 (66)  572 (34) | 378 (62)  230 (38) | 132 (61)  85 (39) | 134 (63)  80 (37) | 184 (60)  121 (40) |
| Age  Mean age, years  (SD)  Range | 39.0  (16.48)  12–84 | 36.8 (15.09)  12–77 | 41.6 (16.70)  12–84 | 43.3 (15.43)  12–77 | 37.9  (16.15)  12–81 | 40.7  (16.12)  12–79 | 46.9 (13.97)  12–76 |
| Study drug exposure, *n*  Total patient years^a^  Mean, days  (SD) | 855  185.59  79.3  (49.21) | 334  87.49  95.7  (48.60) | 1661  1179.36  259.3  (155.05) | 604  169.15  102.3  (59.36) | 214  61.00  104.1  (60.39) | 214  60.28  102.9  (59.16) | 305  95.71  114.6  (60.60) |

^a^Numbers represent the total years of exposure to study drug for all patients in each group

FF/VI 100/25 µg = 92/22 µg (emitted). FF/VI 200/25 µg = 184/22 µg (emitted). FF 100 µg = 90 µg (emitted). FF 200 µg = 182 µg (emitted)

*BD* twice daily, *FF* fluticasone furoate, *FP* fluticasone propionate, *OD* once daily, *SD* standard deviation, *VI* vilanterol
