# Supplementary figures and images for: Integrated safety and efficacy analysis of once-daily fluticasone furoate for the treatment of asthma
Source: Respir Res. 2016 Nov 24;17:157. doi: 10.1186/s12931-016-0473-x (PMC5122018; doi:10.1186/s12931-016-0473-x)

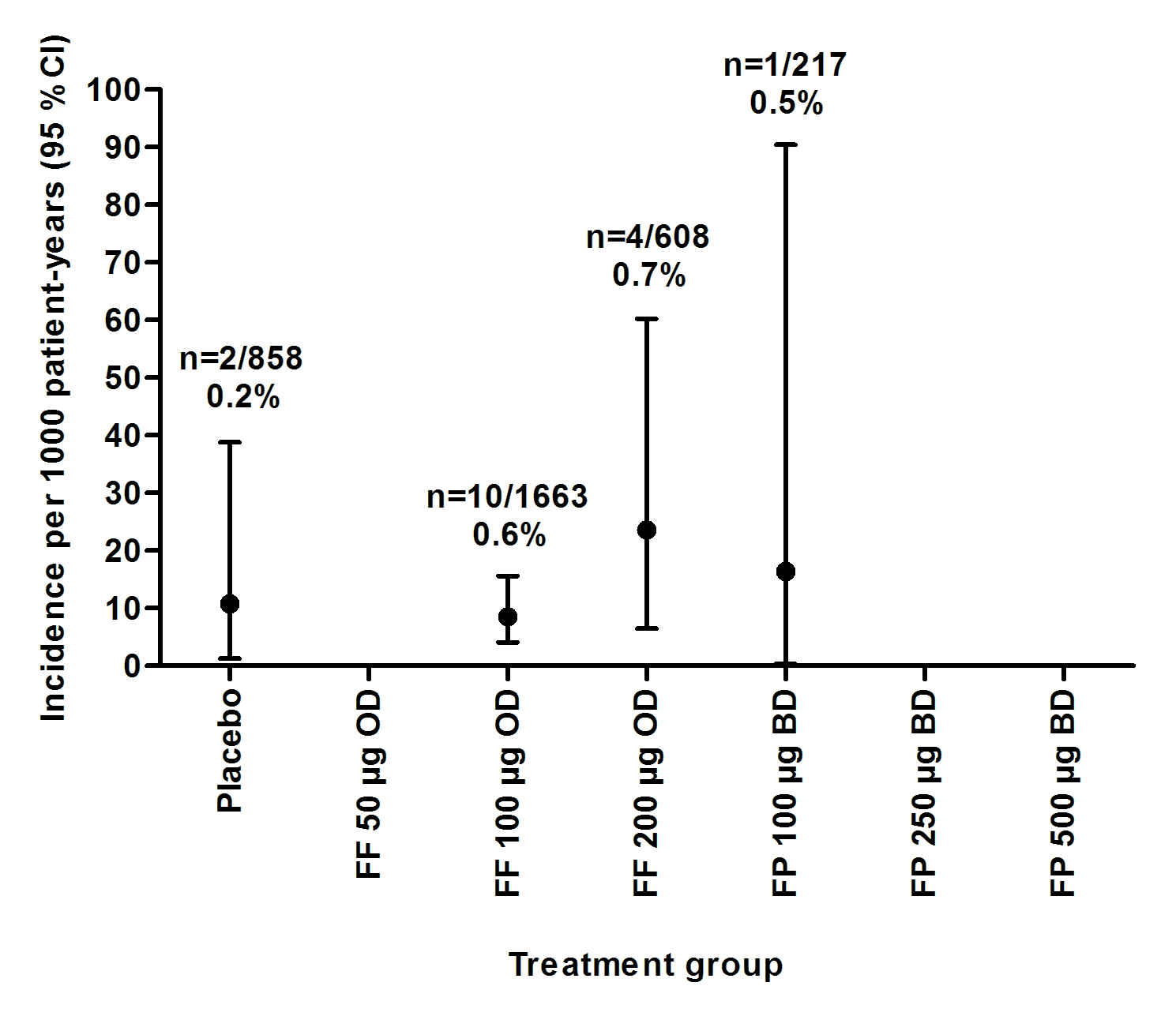

Supplement: Additional file 2: Figure S1. — Plot of pneumonia incidence per 1000 patient-years and 95% CI by treatment group (integrated clinical studies). BD, twice daily; CI, confidence interval; FF, fluticasone furoate; FP, fluticasone propionate; OD, once daily. (TIF 465 kb) [file 12931_2016_473_MOESM2_ESM.tif]

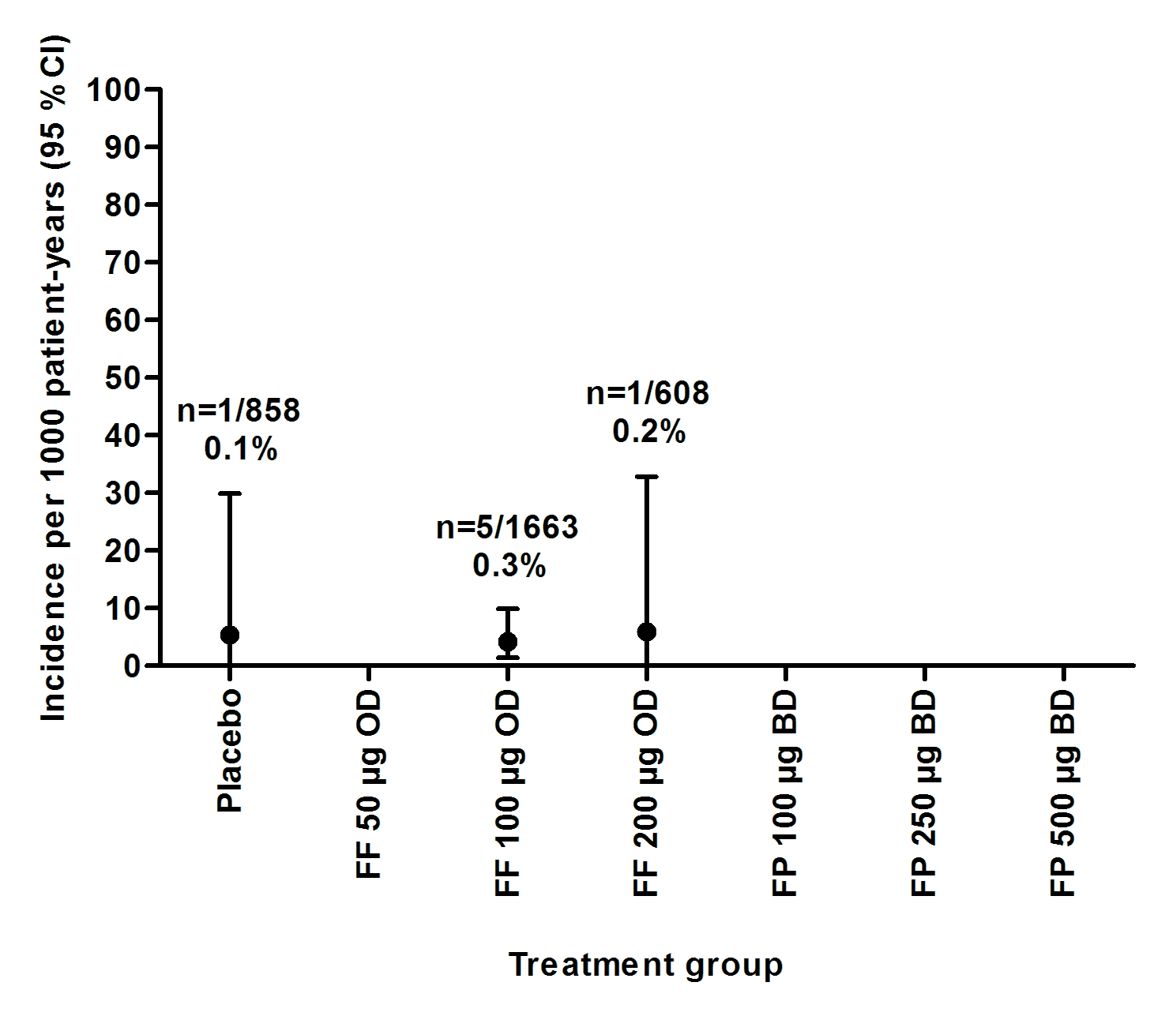

Supplement: Additional file 3: Figure S2. — Plot of serious pneumonia incidence per 1000 patient-years and 95% CI by treatment group (integrated clinical studies). BD, twice daily; CI, confidence interval; FF, fluticasone furoate; FP, fluticasone propionate; OD, once daily. (TIF 427 kb) [file 12931_2016_473_MOESM3_ESM.tif]
